# Recent Advances in the Fabrication and Functionalization of Flexible Optical Biosensors: Toward Smart Life-Sciences Applications

**DOI:** 10.3390/bios11040107

**Published:** 2021-04-04

**Authors:** Bruno Miranda, Ilaria Rea, Principia Dardano, Luca De Stefano, Carlo Forestiere

**Affiliations:** 1 Institute of Applied Sciences and Intelligent Systems, Unit of Naples, National Research Council, Via P. Castellino 111, 80131 Napoli, Italy; bruno.miranda@na.isasi.cnr.it (B.M.); ilaria.rea@na.isasi.cnr.it (I.R.); principia.dardano@na.isasi.cnr.it (P.D.); 2 Department of Electrical Engineering and Information Technology, University of Naples Federico II, Via Claudio 21, 80125 Napoli, Italy; carlo.forestiere@unina.it

**Keywords:** optical biosensors, flexible hybrid materials, disease early-diagnosis, nanofabrication techniques, nanocomposite materials, LSPR-based biosensors, SERS-based biosensors

## Abstract

Over the last 30 years, optical biosensors based on nanostructured materials have obtained increasing interest since they allow the screening of a wide variety of biomolecules with high specificity, low limits of detection, and great sensitivity. Among them, flexible optical platforms have the advantage of adapting to non-planar surfaces, suitable for in vivo and real-time monitoring of diseases and assessment of food safety. In this review, we summarize the newest and most advanced platforms coupling optically active materials (noble metal nanoparticles) and flexible substrates giving rise to hybrid nanomaterials and/or nanocomposites, whose performances are comparable to the ones obtained with hard substrates (e.g., glass and semiconductors). We focus on localized surface plasmon resonance (LSPR)-based and surface-enhanced Raman spectroscopy (SERS)-based biosensors. We show that large-scale, cost-effective plasmonic platforms can be realized with the currently available techniques and we emphasize the open issues associated with this topic.

## 1. Introduction

Optical biosensors have emerged as analytical devices for the rapid [1], cost-effective [2], selective [3], and specific detection of biological compounds (antibodies, nucleic acids, peptides, toxins, etc.), as well as bacteria [4], viruses [5,6], and cells [7]. The specificity of biosensors is an intrinsic property arising from the biorecognition probe immobilized on the surface of the transducing element. To this aim, noble metals nanomaterials represent very efficient transducers, due to their capability of supporting localized surface plasmons (LSPs) [8] and of significantly enhancing Raman scattering of molecules adsorbed onto their surface (SERS) [9].

Localized surface plasmon resonance (LSPR) is the size and shape-dependent coherent oscillation of the conduction electrons of a noble metal, arising when the size of the object is much smaller than the excitation wavelength [10,11,12,13,14]. The excitation of LSPs gives rise to a strong enhancement of the electromagnetic field in the surroundings of the nanoparticles, which makes their resonance locally sensitive to refractive index variations [15]. In particular, silver (Ag) and gold (Au) nanoparticles (NPs) have been studied deeply due to their capability of exhibiting LSPs in the visible region of the spectrum, thus allowing the design of refractive index [16,17] and colorimetric [18,19,20] optical biosensors. When a target analyte is recognized by the nanoparticles, a resonance shift, proportional to the concentration of the analyte, can be measured through UV-vis spectroscopy.

Noble metal nanoparticles immobilized onto a substrate can be used also for surface-enhanced Raman spectroscopy (SERS). SERS is a sensitive and powerful optical technique providing resolutions up to single-molecule detection [21,22]. It has been extensively used for label-free biochemical assays and cell studies [23,24,25]. Two main mechanisms are involved in SERS: the charge transfer between the molecules and the substrate (chemical effect), and the LSPR modes of noble-metal nanoparticles (electromagnetic effect) [26,27]. SERS spectroscopy is performed to collect information about molecular vibrational states, guaranteeing high sensitivity to conformational changes [9]. Metallic nanoparticles provide the selected substrates with strong enhancement factors (EF) of the molecular Raman signals [28]. For SERS spectroscopy, strong efforts have been made to design and fabricate efficient substrates, with enhancement factors of the Raman signals up to 10^14^, to reach ultra-low limits of detection [29].

All the advantages shown by optical devices based on plasmonic nanoparticles have stimulated the continuous improvement of their fabrication techniques. The nanotechnological fabrication processes are based on two main approaches: top-down and bottom-up, which are sometimes combined to obtain a “hybrid approach” [30,31]. While the top-down approach usually requires nanolithographic techniques, which permits the mechanical or chemical etching of the bulk material, the bottom-up approach is based on the chemical synthesis of nanoparticles [32,33,34], starting from “molecular bricks.” In the case of the bottom-up approach some other methods are required to graft the nanomaterials onto the substrates, usually made of rigid materials (glass, silicon, quartz, etc.) [35,36,37].

The concept of flexible optical biosensors has been introduced more recently, due to the necessity of creating some optical platforms capable of adapting to non-planar surfaces, boosted by the advent of flexible electronics [38] and photonics [39]. This property finds its natural application in wearable sensors, conforming to the skin [40,41,42], food-packaging (sensors for food monitoring) [1,43], real-time monitoring of healing processes [44], and 3D cell cultures in scaffolds and organoids (cellular growth rate monitoring) [45]. Other advantages of flexible plasmonic substrates rely on the rapid, real-time, and cost-effective monitoring of a target analyte.

Flexibility allows rapid and high processability, thus extending plasmonic platforms to daily life applications [46]. For these reasons, many researchers have introduced very promising hybrid/nanocomposite transducers, based on the combination of synthetic or natural polymers with metallic nanoparticles. The combination of polymers with optically active nanomaterials generates platforms with extreme ease of integration within microfluidics and microelectronics devices, showing promising developments toward smart and efficient technologies.

Flexible biosensors find unprecedented applications in the design of wearable, point-of-care testing, and food monitoring devices. First, rigid substrates commonly employed for the accommodation of the plasmonic nanoparticles are difficult to employ as wearable sensors since they cannot easily adapt to skin. Also, rigid platforms on the skin could be uncomfortable and they could not find patient’s compliance [47]. Secondly, concerning POCT devices, researchers are moving toward the use of microfluidics to reduce sample volumes and enhance the capability of an analyte to interact with the bioprobe on the sensing surface. In this context, polydimethylsiloxane (PDMS) and poly(methyl methacrylate) (PMMA) represent the gold standards to fabricate microfluidic channels [48,49]. The typical approach to combine microfluidics and rigid plasmonic substrates is the bonding of the two components. The final result is a microfluidic channel having only one wall covered with the transducing element. However, at the microscale, it may be worth having a channel completely covered with plasmonic nanoparticles to increase the detection efficiency and the contact area. While this is not possible with rigid substrates, it can be done with polymeric nanocomposites [50,51]. Finally, in food safety monitoring polymeric optical devices show appealing features to be easily integrated into food packaging, which is mainly involving polymeric materials [52,53].

The elasticity, bending capability, and stretchability of polymers over/in which plasmonic nanoparticles can be impregnated has been opening novel fundamental studies on the coupling mechanisms between plasmonic nanoparticles. This is something that was not feasible with rigid platforms. As an example, the optical response of plasmonic NPs dispersed in a polymeric film can be coupled by compression, due to the reduction of the distance among NPs, or can be decoupled by stretching the polymer [54]. For this reason, flexible nanoplasmonic is rapidly evolving in optomechanics, which combines theoretical physics with optics and material sciences [55,56]. Moreover, these platforms find applications in many other research fields, such as homeland security (i.e., drugs [57] and explosives [58,59] detection), seismology [60], and plant biology [61].

**Figure 1:**
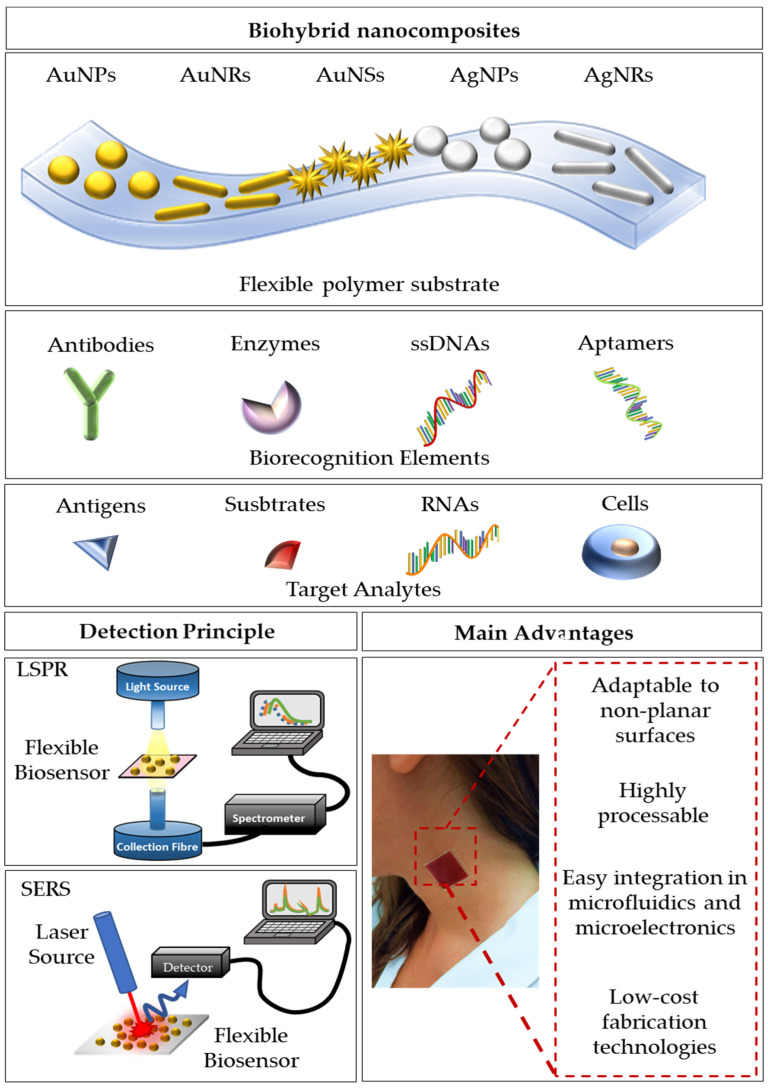
A schematization of flexible optical biosensing platforms reporting the combination of a polymeric substrate with differently shaped gold/silver nanoparticles, the most used biorecognition elements, and target analytes. Furthermore, a schematization of the detection setups for LSPR and SERS signals is reported. Finally, the main advantages are summarized.

Figure 1 briefly schematizes the most used approaches to obtain functional biohybrid nanocomposites, together with the setups usually employed for their optical characterization and the main advantages. The most used nanomaterials are spherical gold and silver nanoparticles, but more complex shapes, such as nanorods and nanostars [29,62,63], are also employed for the fabrication of these optical devices. The shape and the size of the NPs are important design parameters to tune and optimize the optical responses. The biorecognition elements may include antibodies, enzymes, single-stranded DNAs, and/or aptamers, which provide the platform with high selectivity and specificity for the target analytes (antigens, substrates, RNAs, and cells). The LSPR optical setup usually consists of a white light source directly connected to an optical fiber probe. The resonant spectra can be collected in transmission mode if the device is optically transparent, or in reflectance mode, for devices with high reflectivity. A spectrometer is used to collect the transmitted/reflected light. Vice versa, a typical SERS setup consists of a laser source at different wavelengths, whose light is directly conveyed to the devices and collected with a CCD camera to register the Raman signal. In this review, we mainly focus on the description of LSPR-based flexible biosensors and SERS-based flexible biosensors, reporting the most innovative technologies and protocols for the fabrication of bio-responsive materials combining synthetic or natural polymers with gold or silver nanoparticles having diverse shapes and sizes.

Although flexible plasmonic substrate applications are very broad, in this review, we focus on healthcare, food quality safety, and environmental monitoring. We classify LSPR biosensors in 2D and 3D architectures. In 2D architectures, the nanoparticles are arranged on a surface, usually by grafting them on the polymeric substrate. In 3D platforms, the sensing element is all distributed within a volume, typically embedding nanomaterials within their polymeric matrix. We also classify SERS biosensors according to the polymer that is used as a substrate (synthetic or natural). Moreover, for all the reported examples, we focus on the experimental details concerning fabrication and functionalization strategies of flexible plasmonic biosensors. Finally, we also point out the main drawbacks and limitations of the currently available fabrication techniques, together with possible improvements, future perspectives, and their biomedical applications.

## 2. LSPR-Based Flexible Biosensors

The demand for optical biosensors based on LSPR rather than SPR has increased conspicuously in the last two decades. This is mainly due to the different spatial decay of the two sensing platforms. While surface plasmon polaritons (SPPs) exploited for SPR are generated on a thin metallic surface (thickness ~10–250 nm) and have a large spatial decay, localized surface plasmons (LSPs), also known as non-propagating plasmons, are generated on noble-metal nanoparticles, which have characteristic dimensions always well below the excitation wavelength. In the second case, the spatial decay of the electromagnetic field is much smaller and limited to the surrounding of the NPs [64,65,66,67]. This significant difference allows the design of platforms, whose sensitivity is strictly associated with the surface of the NPs and independent from what happens far away from the surface (bulk) [32,33]. In this context, LSPR biosensors show appealing properties such as miniaturization, minimal interferences, and scalable production. However, while both periodic [34,35] and non-periodic [31] arrays of noble-metal nanoparticles on hard substrates have been already proposed as sensing platforms, there is still a lot of active research to propose novel approaches toward the fabrication of flexible, polymer-based LSPR biosensors. The main issue associated with the fabrication of such optical platforms is the limited number of polymers that can be used as substrates. A good LSPR biosensor must be highly transparent or reflective to allow the detection of the optical signal from noble metal nanoparticles. For this reason, opaque polymers, such as nanofibers (commonly employed as substrates for SERS-based biosensors) are not suitable for LSPR sensing.

### 2.1. 2D Flexible LSPR-Based Biosensors

Although a reflectance measurement can be in principle performed to detect LSPR signal from a 2D array of plasmonic nanoparticles, it can become tough when dealing with polymeric substrates. The main reason is that the chosen substrate must be highly reflective to detect a sufficiently noiseless signal; it is the case of silicon wafers and gold layers on glass, as typical examples. A reflective polymeric substrate as a flexible counterpart to hard ones is commonly not easily available and its production requires the use of specific filling agents to confer such a property [68]. For this reason, measurements in transmission mode are usually preferred. Moreover, when dealing with small NPs, the main contribution of the optical response is generally ascribed to absorption rather than scattering, so a reflectance mode setup in LSPR measurements would result in a noisy signal. Besides, this measurement type in flexible biosensors would require a reflecting polymer.

#### 2.1.1. Transparent LSPR Substrates

LSPR signals from noble metal nanoparticles onto transparent flexible substrates, namely absorbance or extinction, are usually monitored via transmission mode setups (as schematized in Figure 1). This measurement methodology requires high transparency from the sample substrate. Polydimethylsiloxane (PDMS), an organosilicon compound that has been widely used to realize microfluidic devices, as well as medical devices, represents the ideal candidate due to its low-cost, high transparency in UV-Vis spectral range, and the abundance of processing techniques that have been extensively reported elsewhere [33,38,39,69,70].

**Figure 2:**
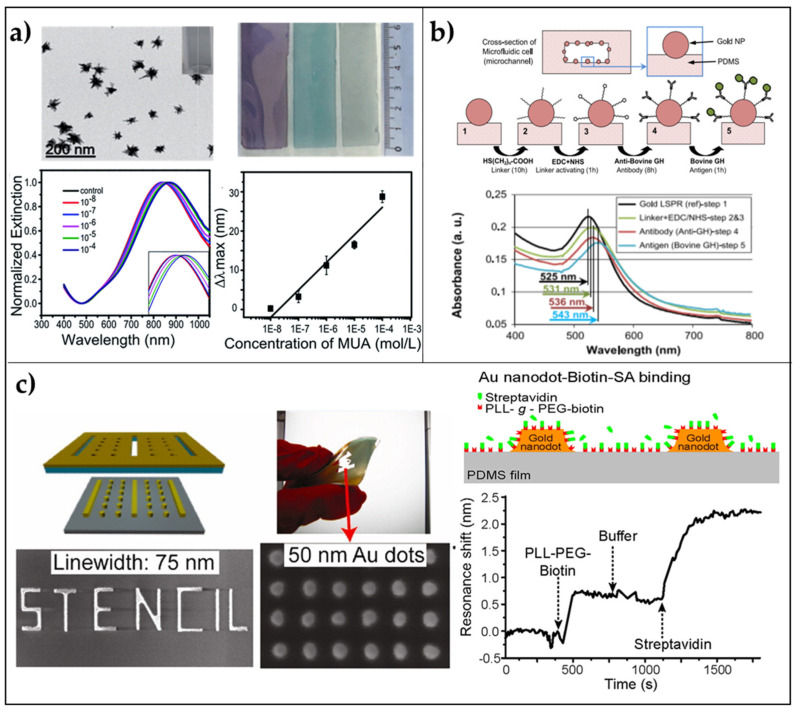
2D arrays of AuNPs on PDMS flexible substrates. (**a**) Immobilization of AuNSs on PDMS: TEM images of gold nanostars (scale bar 200 nm); macroscopic morphology of PDMS/AuNSs platforms; extinction spectra of a PDMS/AuNSs film immersed in different concentrations of MUA in ethanol; LSPR shift versus MUA concentration. Reproduced from [71] with permission from Royal Society of Chemistry Pub. (**b**) In situ growth of AuNPs in a microfluidic chamber: Biosensing experiments performed by using the annealed microfluidic biosensor (400-2 cells) prepared from 2% aqueous solution of the gold precursor (48 h); cross-section of a microchannel and AuNPs in the channel; four steps of the biosensing protocol; a legend of the schematics; Au-LSPR corresponding to the four sensing steps, and LSPR band shift corresponding to different Ag concentrations. Reproduced from [72] Copyright (2013), with permission from Elsevier. (**c**) Patterning via Nanostencil Lithography: Schematics of biosensing experiments with nanodots on PDMS. Au nanodots and PDMS are biotin-functionalized, and then streptavidin binds to biotin. LSPR shift of an array of W = 75 nm and S = 50 nm Au nanodots on PDMS upon the addition of biotinylated molecules and streptavidin. The arrows indicate the addition of biomolecules and buffer rinsing. Adapted from [73]. Copyright (2012) with permission from American Chemical Society.

As a first example, we discuss the very simple fabrication of an LSPR platform based on the electrostatic self-assembly of gold nanostars (AuNSs) onto PDMS, reported by Shiohara and coworkers [71]. PDMS slabs were fabricated starting from the elastomer pre-polymer solution with its curing agent (elastomer and curing agent are used in a 10:1 *w*/*w* ratio) poured onto a glass slide. Heating at 60 °C overnight was required to cure the solution. The freshly prepared slab’s surface was modified with (3-Aminopropyl)triethoxysilane (APTES) at 40 °C for 4 h, and finally, soaked in AuNSs solution overnight. The authors tested the refractive index sensitivity of the sensor with three different populations of gold nanostars, and as expected, the highest sensitivity of 392.5 nm/RIU was achieved for nanostars having the longest tips (tip-shape effect) in the range 1.3334–1.4318. The sensing capability of this platform was assessed by measuring the LSP resonance shifts as a function of increasing mercaptoundecanoic acid (MUA) concentrations (from 10^−8^ M to 10^−4^ M), achieving a LOD of 10^−7^ M (Figure 2a). This very first result was very promising due to the very small size of the target molecule. Therefore, greater results in terms of shift and sensitivity could be achieved with bigger molecules such as antibodies.

A possible strategy to obtain a stable grafting of the AuNPs onto PDMS is to perform an in situ reduction of chloroauric acid (HAuCl_4_) onto the already prepared PDMS substrate. This is the strategy followed by SadAbadi and coworkers that proposed a microfluidic chamber with plasmonic nanoparticles for the detection of growth hormones [72]. Exploiting microfluidics in biosensing has attracted the interest of many research groups due to the capability of these tiny channel chambers of reducing the sample volumes with high-throughput and low costs for fabrication [74,75]. SadAbadi and coworkers prepared gold NPs directly on PDMS microfluidic channel showing slower reaction times compared with macro-scale synthesis, which led to the narrower size distribution of AuNPs within the channel (120–130 nm). Such a platform was used to detect the growth hormone achieving a LOD of 3.7 ng/mL (185 pM). This result demonstrates the appealing properties arising from the combination of polymers with plasmonic nanoparticles (Figure 2b).

#### 2.1.2. Periodic 2D LSPR Substrates via Stencil Lithography

Stencil lithography is a relatively recent technique, which is based on the patterning of rigid and polymeric substrates using parallel shadow masks. The major advantage of such a technique is the reusability of the stencil masks that reduce nanofabrication costs. Moreover, no thermal or photoresists, nor reactions, chemical solvents, or mechanical pressures are required when dealing with very fragile polymers. With this technique, a resolution down to few tens of nanometers has been achieved in the fabrication of metallic nanodots and nanowires [73]. In the same work, the authors proposed an array of nanodots (mean size ~75 nm, pitch ~50 nm) on PDMS showing promising results in terms of biosensing capability. The biotin-streptavidin interaction was successfully determined by extinction measurements exhibiting a plasmon resonance shift of 1.7 nm after exposure of the substrate to a streptavidin concentration down to 20 μg/mL (Figure 2c).

### 2.2. 3D Flexible LSPR-Based Biosensors

Most of the research in biosensing platforms moves toward the design of 2D surfaces with biorecognition elements on the top. The amount of probes that can be efficiently immobilized on the transducing element is strictly dependent on the surface area and surface-area-to-volume ratio, which are limited when dealing with 2D devices. Therefore, planar surfaces often show evident limitations, such as instability of the immobilized probes, narrow dynamic range, low LODs as a direct consequence [76]. The relevance of surface area and surface-area-to-volume ratio to boost the sensitivity of a platform toward a target analyte, as well as the response time, was recently assessed by Barbosa and coworkers [77]. In this context, miniaturized 3D biosensors offer promising opportunities since the 3D architecture and configuration of the transducing element on which the bioprobe is immobilized significantly enhances the available surface area and surface-area-to-volume ratio enabling higher analytical performance. This is especially true when the transducing element is a nanomaterial with optical properties such as noble metal nanoparticles to design LSPR biosensors. Polymeric materials represent the ideal candidates to design optical biosensors with a 3D architecture since their characteristic dimensions can be tuned from the nanometer scale to the mesoscopic scale with a large variety of techniques such as UV-photolithography, 3D bioprinting, and stencil lithography, among the others.

#### 2.2.1. Periodic 3D LSPR Substrates via Nanosphere Lithography

**Figure 3:**
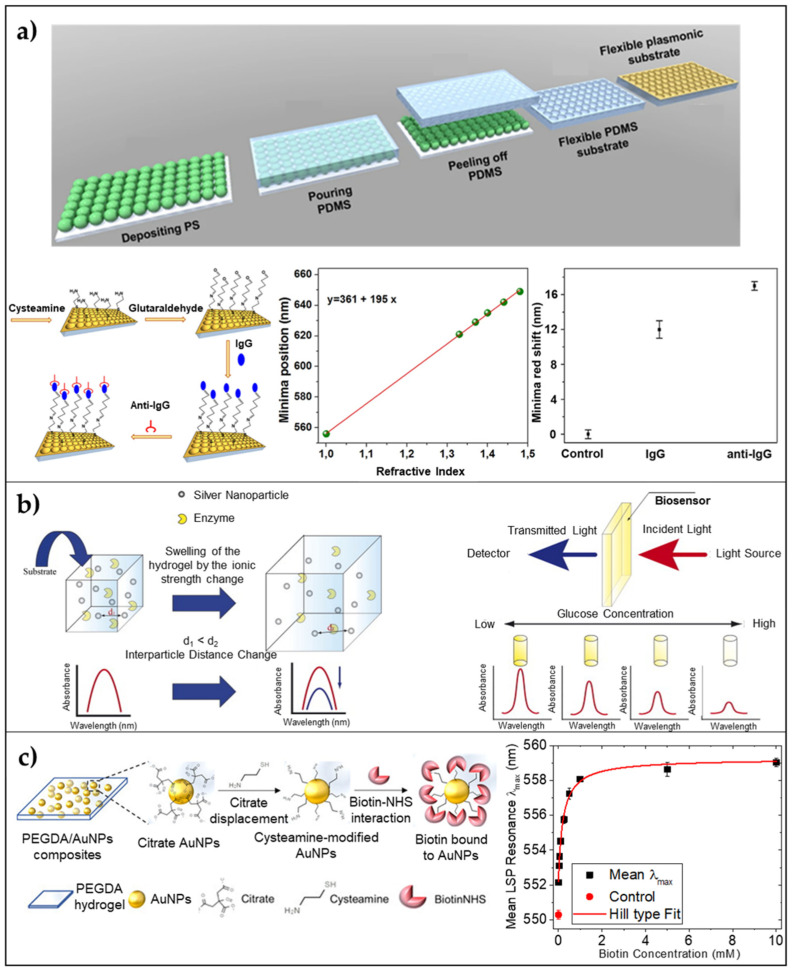
3D arrays of AuNPs on/embedded in flexible substrates/matrices. (**a**) 3D Nanocups on PDMS: Schematic illustration of the fabrication steps of the flexible 3D Au nanocups platform; sensitivity measurements leading to a value of 195 nm/RIU; and reflectance response after human IgG immobilization and specific anti-human IgG detection Adapted from ref. [80] Copyright (2017) with permission from Springer Nature. (**b**) Stimuli-responsive hydrogel embedding AgNPs: Schematic illustration of detection principle of LSPR-based optical enzyme biosensor. Reproduced from Ref. [81] Copyright (2008), with permission from Elsevier. (**c**) PEGDA hydrogel nanocomposite embedding AuNPs: Schematics of the functionalization procedure involving citrate displacement with a cysteamine modification of AuNPs and the subsequent sulfo-NHS biotin grafting on the available amino groups of cysteamine within the nanocomposite, and LSPR λ_max_ (black squares) as a function of the biotin concentration (from 25 μM to 10 mM). Adapted from [17] Copyright (2021), with permission from AIP Publishing.

Analogously to 2D periodic LSPR flexible sensors, the achievement of ordered 3D plasmonic nanoarrays on a flexible substrate is still an active research field, especially when the common goal is to give rise to high-performance LSPR devices with limited fabrication costs. To this aim, several techniques have been proposed, but most of them require expensive and time-consuming master molds [78]. Replica molding is currently used in many research areas and a lot of efforts have been made to produce low-cost molds on/in which plasmonic nanostructures could be arranged. In this frame, nanosphere lithography, combined with soft lithography can be used to fabricate large-area devices by using small spherical particles to obtain a template for lithography. On this templated substrate, a sputtering of a gold or silver thin layer can be performed to achieve a 3D array of nanostructures [79]. Inspired by this approach, Focsan et al., proposed a novel strategy to fabricate a large-scale, flexible, and tunable 3D gold nanocups platform for LSPR sensing [80]. Briefly, PDMS elastomer was used for pattern replication of a closely packed monolayer of polystyrene nanoparticles. After the PDMS curing a coating of gold was deposited on the PDMS obtaining a large-area of nanocups array. Different sizes of PS particles were used to tune the optical response of the device. To test the LSPR platform sensing performance, a sensitivity measurement was carried out on the platform obtained starting from a PS size of 719 nm, and a sensitivity value of 195 nm/RIU was achieved. Moreover, human IgG (1 mg/mL) was immobilized onto the surface and an anti-human anti-IgG target (1 μg/mL) was detected via reflectance measurements causing two red-shifts of 12 and 5 nm, respectively (Figure 3a). This platform was used also as a SERS substrate, showing promising results also in this case.

#### 2.2.2. 3D Nanocomposite Hydrogels

Hydrogels are regular networks obtained from the crosslinking reaction of specific polymer chains. They have been extensively used in biomedical applications, ranging from tissue engineering [82,83] to drug delivery [84,85,86]. Their capability of absorbing a big amount of water (up to 90% of their volume) arises from the strong hydrophilic nature of the polymeric chains. Recent studies have demonstrated their potential as 3D immobilization matrices of plasmonic nanoparticles for their intrinsic properties. Hydrogels are usually anti-fouling, biocompatible, and biodegradable; moreover, they preserve the activity and functionality of biomolecules within their network, thus showing the ease of integration into complex micro-systems [87,88]. For these reasons, the term “bio-responsive hydrogels” was introduced to describe a 3D polymeric network that produces a detectable response after interaction with a biochemical compound. The crosslinking reaction can be performed through several methodologies, including the use of cross-linkers, polymer-polymer binding, photo-active agents, or enzymes. However, photopolymerization is one of the most used approaches in the soft lithography of nanocomposite hydrogels.

Plasmonic nanoparticles can be embedded within a hydrogel by sampling mixing a colloidal suspension of NPs with a pre-polymer solution in convenient volume ratios. For example, Randriantsilefisoa et al. [89], recently reported proof of concept showing the ability of polyol-based hydrogels to exhibit a strong colorimetric variation and shrinkage in the presence of a certain concentration of influenza A virus. Briefly, they start from a pre-polymer solution of dendritic polyglycerol cyclooctyne (dPG) with a molecular weight (MW) of 10 kDa, and diazide-poly(ethylene) glycol (PEG) with a total MW of 6 kDa. The two polymers tend to form a hydrogel using a strain-promoted alkyne–azide cycloaddition (SPAAC) reaction. The hydrogel was polymerized with sialic acid stabilized AuNPs showing high specificity and affinity with the hemagglutinin (HA) protein present in influenza A virus (IAV) in a multivalent manner. For such a reason, the detection of the virus was possible by simply looking at the colorimetric variation of the hydrogel. Analogously, a quantitative LSPR response from plasmonic nanoparticles embedded in a hydrogel matrix was reported by Endo ad coworkers [81]. They fabricated an LSPR enzyme biosensor using a nanocomposite of polyvinyl-pyrrolidone (PVP) hydrogel and silver nanoparticles functionalized with glucose oxidase enzyme (GOx). Due to the high affinity of GOx for glucose, upon the soaking of the platform in a glucose solution, a decrease in the absorbance spectrum of AgNPs was observed due to the swelling of the polymer proportional to the biomolecule concentration. This phenomenon was easily explained by the increase in the distance between NPs. The presence of the anionic reduced form of FAD in the reaction of GOx with glucose produced an enhancement in the swelling capability of the hydrogel, which strongly affected the scattered spectrum of the nanocomposite. The proposed sensor was able to detect glucose concentrations down to a LOD of 10 pM, showing great potential as a cost-effective and highly sensitive platform for medical applications (Figure 3b). More recently, Miranda et al., proposed the design and a large-scale, low-cost fabrication strategy of a poly(ethylene) glycol diacrylate (PEGDA) hydrogel embedding size-varying citrate AuNPs [17,90]. They show how the hydrogel physically retains spherical AuNPs within the mesh network allowing increased stability of AuNPs. A sensitivity of 110 nm/RIU in a range of refractive indices from 1.33 and 1.48 is reported together with a direct surface modification of AuNPs within the mesh network leading to the detection of Biotin down to a 25 μM concentration (Figure 3c).

## 3. SERS-Based Flexible Biosensors

SERS optical biosensors leverage on the design and fabrication of periodic, quasi-periodic, or random metal nanostructured arrays (nanohole arrays [91,92,93], nanocanals [94], porous structures [95,96,97,98,99,100], etc.) on rigid substrates (alumina, silicon, glass, etc.), showing very efficient performances, in terms of sensitivity and limits of detection. However, for the SERS measurements, the adsorption of the analyte of interest onto the surface is a necessary step, which is not always straightforward. It requires the extraction and the collection of the biomolecule and the selection of suitable surface chemistry for the successful binding of the analyte onto the substrate [101]. To obtain efficient and fast in situ detection, a rigid and opaque substrate may limit the applications to planar surfaces.

For this reason, the development of flexible, transparent substrates is very promising to overcome these issues, allowing the non-destructive detection of the target analytes. Among the currently used materials, we can distinguish between synthetic and natural polymers as SERS flexible substrates, whose performance is comparable to the previously mentioned rigid platforms [102,103].

### 3.1. SERS-Based Biosensors with Synthetic Polymers

#### 3.1.1. Polymeric Nanofibers

A class of substrates that have been extensively used for flexible SERS substrates is certainly one of the polymer (nano)fibers. They can be impregnated or sputtered with metallic nanoparticles using low-cost techniques. Large moieties of free-standing polymer fibers can be made by electrospinning [46]. It is possible to spin a pre-polymer solution containing metal nanoparticles, such as Ag nanoparticles [102] or Au nanorods [103], within a poly(vinyl alcohol) (PVA) matrix, in such a way that the particles will align along the fiber direction. Alternatively, previously spun fibers can be sputtered with thin metallic films or nanoparticles. For example, it is possible to deposit Ag nanoparticles on a pre-functionalized poly-acrylonitrile (ASFPAN) by in situ and seed-mediated growth [104]. However, to achieve reproducible SERS signals, for the detection of a target analyte it is important to achieve a semi-ordered arrangement of the nanoparticles and avoid aggregates that could negatively affect the reproducibility of the substrate. Many strategies have been implemented for this goal (i.e., electroless deposition, non-covalent and covalent interactions), including the introduction of functional groups having a high affinity for metallic nanoparticles [105].

**Figure 4:**
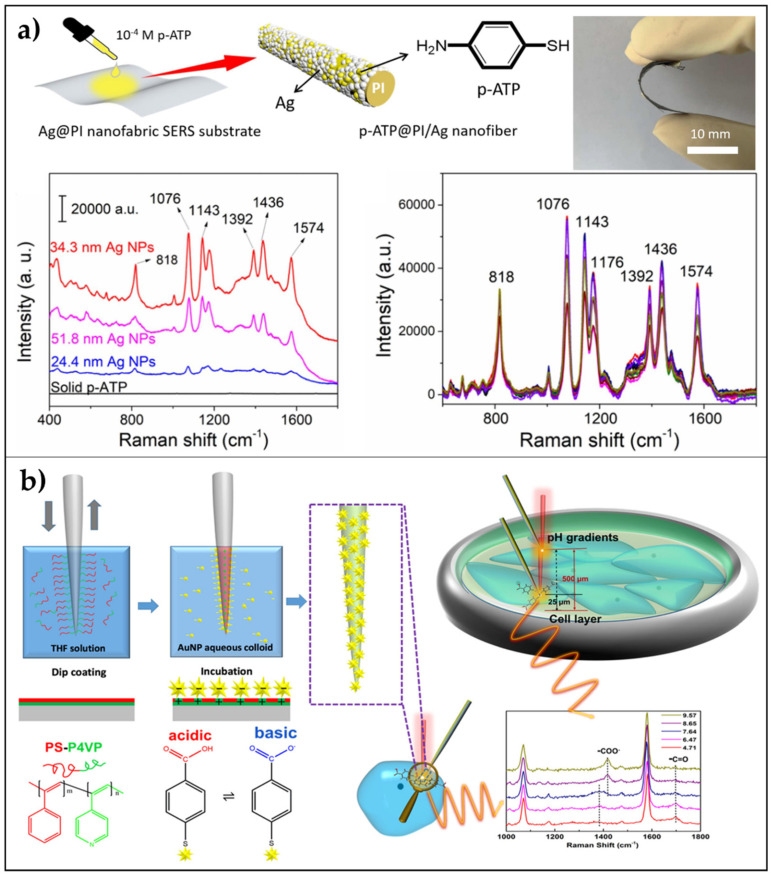
Nanofibers as flexible SERS substrates. (**a**) AgNPs on PI nanofabric: A schematic diagram showing the preparation of the sample for SERS characterization, SERS spectra of the solid p-ATP powder, and the 1.0 × 10^−4^ M p-ATP dropped on the surface of the Ag@PI nanofabric (5 μL on 9 mm^2^) with different diameters of Ag nanoparticles (24.4 nm, 34.3 nm, and 51.8 nm), and SERS signal reproducibility of the p-ATP on the Ag@PI nanofabric with 34.3 nm Ag nanoparticles recorded on different randomly selected spots. Reproduced from [106] Copyright (2020), with permission from Elsevier. (**b**) Branched AuNPs on PS-P4VP nanofiber: uniform distribution of well-dispersed branched AuNP arrays, which allow for significantly higher SERS response than spherical AuNPs, and schematic representation of the fabrication procedure with application in the direct measure of the internal pH of cells and external pH gradients in cell culture environments. Adapted with permission from [107] Copyright (2020) American Chemical Society.

In this context, Kong et al. [106], polyimide developed an Ag@polyimide (PI) nanofabric SERS-active substrate by integrating electrospinning strategy and ion-exchange in situ reduction process and using DMAB (dimethylamine-borane) as reducing agent. The use of this agent allowed the uniform distribution of AgNPs on the surface of nanofibers, yielding a flexible nanofabric, and the size of the Ag NPs could be easily regulated by adjusting the DMAB concentration. P-ATP was selected as a target analyte to demonstrate the high SERS sensitivity and reproducibility of the substrate, which showed an EF of about 9.0 × 10^3^ and a LOD of 10^−14^ mol/L (Figure 4a).

Moreover, Saravan et al. [108] reported on the synthesis of electrospun nanofibers starting from a blend of modified thiol functionalized adenine (L) and PAN polymer. The AuNPs/PAN/L fibrous mats had an AuNPs concentration, which could be directly related to the percentage of the bioessential ligand available on the surface. The realized flexible substrate was used for sensing uric acid, whose clinically relevant concentrations fall in the range 10^−3^–10^−4^ M. The authors reported a LOD of 10^−7^ M with an EF of 10^4^.

Recently, Zhao et al. [107], combined the low-invasiveness and high spatial resolution of nanofibers and branched AuNPs (nanostars and nanoribbons) arranged in a monolayered distribution into a reproducible SERS biosensor. The authors reported the spatial mapping of the pH gradient in a cellular environment with applications in cancer diagnosis and non-invasive cell monitoring. The greatest sensitivity was achieved by nanofibers covered with AuNSs with short tips (68 ± 15 nm). The fabrication procedure can be summarized into two main steps: dip-coating of the nanofiber in tetrahydrofuran (THF) solution of PS-P4VP (poly(styreneb-4-vinylpyridine)) and incubation of the brush-layer coated nanofiber in a colloidal solution of AuNSs for 3 h.

The first step generates a coating of the fiber with an irreversibly adsorbed brush layer, which allows the formation of a monolayered distribution of unaggregated gold nanoparticles (second step) [109]. To poly(styrene) (PS) is attributed the capability of forming a corona around the AuNPs that could sterically prevent the aggregation. The high reproducibility and stability of the SERS sensor were confirmed by statistical analysis (relative standard deviation below 7%) in the pH linear range from 6.5 to 9.5. Such sensitivity allowed the determination of the intracellular and extracellular pH of breast cancer cells. Moreover, the pH spatial distribution in a monolayer of MDAMB-231 cells was measured confirming the appealing properties of such a device in biomedical applications toward cancer early diagnosis and therapy with minimal invasiveness (Figure 4b).

#### 3.1.2. Transparent Polymers

Flexible and transparent SERS substrates can be realized through the combination of noble-metal nanoparticles and polydimethylsiloxane (PDMS). PDMS films, showing a very high optical transparency, can be easily fabricated, and used as support for metallic nanoparticles. Spherical AuNPs and AgNPs have been immobilized and embedded into PDMS elastomer by several research groups.

For instance, Lu et al. [110] reported the coating of spherical AuNPs and AgNPs on an (APTES)-modified PDMS elastomer and they used the realized SERS substrate for the sensitive detection and imaging of micropatterns of methylene blue and p-aminothiophenol. However, the poor adhesion of the nanoparticles on the PDMS film may limit their application: in fact, the functionalization of the nanoparticles with a probe often requires many chemical steps that can loosen the nanoparticles anchoring to the surface, thus varying their surface density. Moreover, spherical nanoparticles provide weak SERS signals, making it impossible to reach ultra-low limits of detection. For these reasons, many efforts have been done, on the one hand, to synthesize sharp nanoparticles to efficiently enhance the SERS signals [71], on the other hand, to provide chemical and mechanical stability at the interface between the polymer and the nanoparticles.

**Figure 5:**
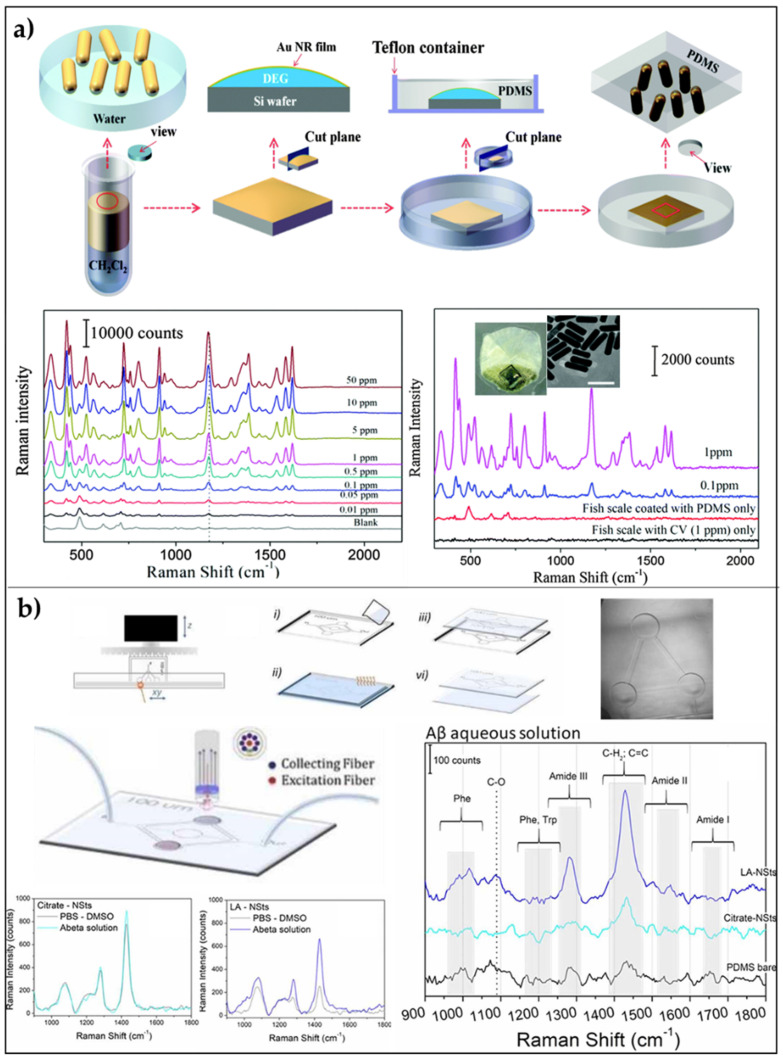
SERS platforms based on transparent polymers: (**a**) AuNRs/PDMS platform: schematic illustration of the preparation of AuNRs/PDMS substrate, SERS spectra of aqueous CV at various concentration, and SERS spectra of CV acquired by applying the nanocomposite film on the fish scale and then illuminating from the backside with a 785 nm laser. Adapted with permission from [101]. Copyright (2017) American Chemical Society. (**b**) AuNSs/PDMS microfluidic channel for SERS: Schematic illustration of the fabrication process of depicting the final device coupled with the Raman fiber probe; Raman spectra of aβ-42-peptide against buffer solution for Citrate-AuNSs substrates, and LA-AuNSs-PDMS; finally, a comparison of the background-subtracted Raman ‘fingerprint’ spectral region of aβ-42-peptide between bare PDMS, Citrate-AuNSs, LA-AuNSs substrates. Adapted with permission from [50]. Copyright (2020), IOP Publishing Ltd.

Park et al. [101], successfully introduced an ultra-high sensitive SERS substrate based on assemblies of gold nanostars (AuNSs) embedded into PDMS films. First, they dip-coated AuNSs onto a poly(diallyl-dimethylammonium) (PDDA)-modified silicon substrate, exploiting electrostatic interaction. Then, they poured PDMS pre-polymer solution, and, after curing it at room temperature, they slowly detached the PDMS/AuNSs assembly from the Si substrate. Through this fabrication technique, it is possible to obtain a uniform coating of the PDMS. To test the efficiency of the realized substrate, benzenethiol molecules were immobilized on different substrates (silicon, glass, Ag film, and Au film) as a comparison. They report an enhancement factor of ≈1.9 × 10^8^ and sensitive detection of analytes down to 10^−8^ M. Alternatively to electrostatic interaction, a gel-trapping fabrication technique was proposed and combined with liquid/liquid interface self-assembling of gold nanorods (AuNRs), chosen for their strong LSPs in both transversal and longitudinal directions [111]. PDMS pre-polymer solution was used to entrap the NRs at the interface between the two immiscible liquids. The sensing capability of AuNRs PDMS platform was tested on crystal violet (CV) in aqueous solutions and a concentration down to 10 ppb with EF = 0.87 × 10^5^ was achieved (Figure 5a). Moreover, the signal uniformity RSD was assessed at 3.9%. In the same context, a relatively simple technique to fabricate plasmonic Ag Nanocubes (NCs) on PDMS was introduced as a SERS platform starting from an organic/water interface between cyclohexane and water. This technique exploits the so-called zero-transferring, i.e., the capability of preserving the spontaneous nanoparticles arrays formation at the interface between two immiscible liquids [112]. PDMS was directly inserted at the interface and no rigid materials were used for the fabrication of this device. Plasmonic Ag Nanocubes arrays onto PDMS were used as SERS substrate, producing enhancement factors of ~3.43 × 10^6^ and LODs 10^−10^ and 10^−9^ M for methylene blue (MB) and rhodamine 6G (R6G), respectively. The designed platform yielded a good reproducibility (RSD ~12%) and a good selectivity versus the different target analytes. The main advantage of these fabrication strategies lies in the capability of keeping intact the nanocubes arrays since no transfer from a substrate to another one is required, thus preserving the great number of hotspots on the PDMS surface. The potential application of this platform was tested in food analysis. In particular, the detection of CV contamination in fish down to a concentration of 0.6 ppm was performed directly on the fish skin, by acquiring the backscattering from the reverse side of the substrate.

The greatest and more technologically advanced evolution of flexible SERS substrate based on PDMS is the implementation of this technology within microfluidic systems. Dallari et al. [50] recently reported a novel SERS-microfluidic prototypes combining PDMS replica molding with advanced 3D printing and gold nanostars for the detection of Aβ-42-peptide (Aβ), considered one of the main pathological biomarkers for Alzheimer’s disease. The demonstration of the potential applications of the platform in diagnostics is made by testing a 4 µM aqueous solution of Aβ-42-peptide (Aβ). The Raman spectra reported in Figure 5b show that the Raman signal intensity of Aβ is higher in presence of metallic nanostructures, while it is almost non-visible on bare PDMS (Figure 5b).

### 3.2. SERS-Based Biosensors with Natural Polymers

The quest for “green” and more eco-friendly biosensors arises from the negative environmental impact of plastics and synthetic materials, commonly used for the design of flexible biosensors. This necessity has stimulated the interest of many research groups toward novel SERS optical platforms based on natural materials. For example, natural biosilica-based SERS responsive devices have been recently reviewed by Tramontano et al. [113]. In this context, natural polymers represent valid alternatives for flexible eco-friendly SERS platforms [114].

Particularly, Turasan et al., [115] used electrospinning to create a new zein-based SERS biosensor platform. Zein is a class of prolamin proteins found in maize (corn). Electrospun zein-nanofibers enabled the production of a substrate with high surface area and roughness, reduced amount of gold nanoparticles for the fabrication, and good biodegradability of the sensor. To form zein-nanofibers the authors chose acetic acid as a solvent in which 26 wt.% of zein was dissolved. The resulting nanofiber diameter was 289 nm. After 12% glutaraldehyde crosslinking, a drop deposition method was used to cover the surface with gold nanoparticles. The Raman signal of rhodamine 6G was monitored registering an enhancement factor of 1.06 × 10^6^. The promising achievement that the authors declare is associated with the use of gold: 803 times less gold (by weight) was used with respect to ref. [114], offering a greener and more eco-friendly alternative to plastic-based SERS devices. However, such a device has not yet been exploited for biosensing applications.

On the contrary, Asgari et al., [116] proposed a cellulose/Au@Ag nanocomposite, as a SERS platform for the detection of pesticides (thiram and paraquat). Cellulose is one of the most abundant natural polymers on earth that has gained attention due to its intrinsic properties, ease of functionalization, and biodegradability [117]. The natural wrinkles and high porosity of cellulose combined with plasmonic nanoparticles result in a high SERS sensitivity. The use of nanofibrillar cellulose (NFC) resulted in an improvement of the homogeneity of the NPs distribution. Au@Ag NPs were obtained by bottom-up chemical reduction of Ag in a solution of Au seeds. The NFC/Au@Ag NPs nanocomposite was fabricated by air-drying at room temperature a mixture 1:1 of NFC suspension and concentrated Au@Ag NPs. The authors performed a concentration analysis for both thiram, a pesticide whose usage is forbidden in lettuce cultivations, and paraquat an herbicide with toxic effects upon a threshold concentration, recording their SERS spectra onto the freshly prepared substrates. LODs of 71 μg/L and 46 μg/L were achieved for thiram and paraquat, respectively.

## 4. Promising Applications of Flexible Biosensors

### 4.1. Point-of-Care Testing for Disease Diagnosis

**Figure 6:**
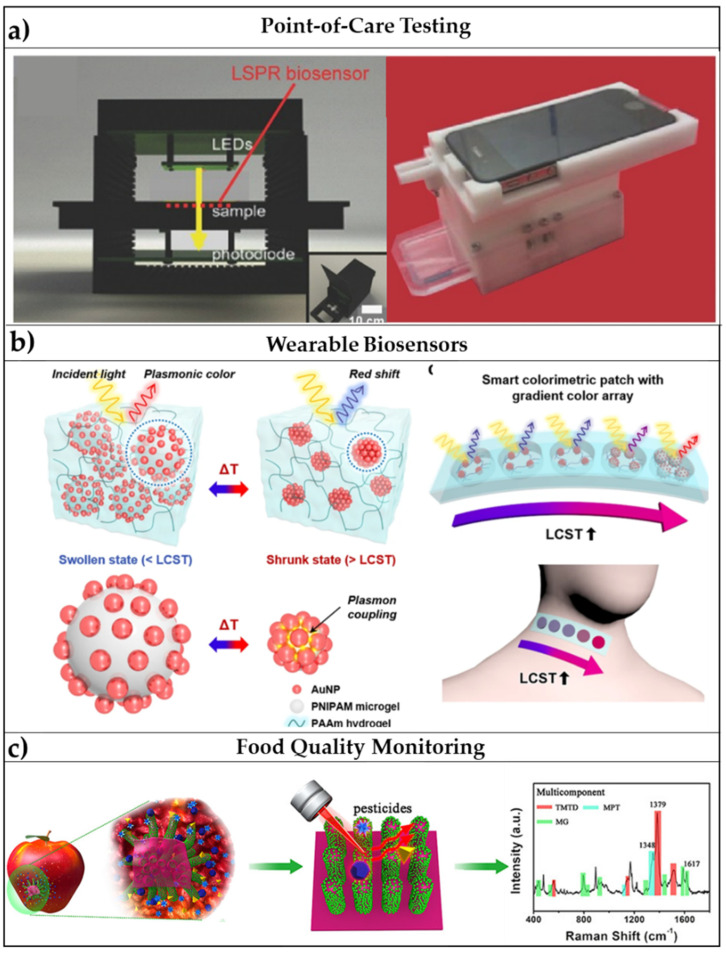
Promising applications of flexible optical biosensors: (**a**) POCT devices: schematic illustrations of smartphone-integrated, microfluidic channel-integrated, and miniaturized optical components-integrated LSPR platforms. Adapted with permission from [121]. Copyright (2017) John Wiley and Sons. (**b**) Wearable sensors: Schematic illustration of plasmonic thermo-responsive microgels under swollen and shrunk states with inset images of the sensor arrays attached to neck and hand. Adapted from [124] Copyright (2018) with permission from Springer Nature. (**c**) Food quality monitoring: Schematic representation of a SERS-based flexible biosensor for the monitoring of pesticide residues on vegetables and fruits. Adapted from [125], Copyright (2017) with permission from American Chemical Society.

There is an increasing demand for portable biosensors, where the clinical diagnostics is directly transferred from equipped laboratories to the patient on site-diagnosis. This need asks for renovated fabrication strategies of point-of-care testing (POCT) devices, which show ease-of-use, compact size, and limited costs [118,119]. Many examples of already commercialized POCT have been reviewed recently and include pregnancy tests, glucose testing, and HIV testing [120]. LSPR- and SERS-based flexible biosensors are promising transducers for the design of a POCT due to the ease of integration with microelectronics and microfluidics [121] (Figure 6a).

A first example of the integration of an LSPR platform with microfluidics has been reported by Huang et al. [122]. They introduced an approach to continuously monitoring the light transmission from an array of AuNPs arranged in a microfluidic channel. A green LED was used in substitution the typical halogen light source. The authors reported a sensitivity of 10^−4^ in RIU. The sensing capabilities of the proposed biosensor were shown by measuring the absorbance variation arisen from biotin/anti-biotin interaction. A LOD of 270 ng/mL was successfully achieved. This first example highlights the importance of the design process of both microfluidics and miniaturized optical components. More precisely, microfluidic channels must be highly transparent, to be compatible with light pathways, they should ensure an efficient sample delivery and minimize reagents and sample consumption [119]. On the other side, spectrometers and light sources (optical components) must be miniaturized to obtain a compact device and, although this is often not very easy, some methods to integrate LEDs for the transducer illumination and miniaturized spectrometers for the collection of the signal have been already proposed to overcome this issue [120]. POCTs for the diagnosis of disease especially in developing countries, where expensive laboratory equipment and specialized operators are not easily available are crucial for the rapid screening of a population. In this scenario, the low-cost polymers-based plasmonic devices offer the possibility to extend the modern lab technologies all over the world and give the less well-off the possibility to access to fast diagnosis and appropriate health care [123].

### 4.2. Wearable Sensors for Rapid Pre-Screening

The design of wearable biosensors for the early diagnosis of diseases has seen many efforts in sensors research. Unfortunately, these novel platforms generally suffer from low reproducibility in sensing capabilities as well as a lack of accuracy in the robust quantification of biomarkers from the skin due to the very tiny concentrations and species of targetable analytes in sweat. Moreover, some crucial issues are still topics of active research: data acquisition, processing, power supply, adaptability to non-planar surfaces (e.g., skin) [42]. Of course, sensitivity, selectivity, and low limits of detection are crucial in any sensing platform, but, in the case of wearable sensors, the collection of skin fluids from the body in a non-invasive way is still an open challenge. Some attempts involving textiles and hydrogels for their absorbing capability have been proposed. However, these materials are not suitable for the precise control of the collected volume.

The combination of micro-and nano-technology for flexible plasmonic biosensors has given rise to platforms with integrated functions all focusing on a single device having a few-millimeters size. In these cases, microfluidics and microelectronics can be combined with flexible plasmonic platforms to produce wearable optical biosensors, whose readout can be performed to the naked eye or via integration with smartphones [41]. Wearable optical biosensors find their potential applications in the fast screening of the population for the detection of a target pathogen, which, in the era of SARS-CoV-2 (severe acute respiratory syndrome coronavirus 2), have revealed as crucial to avoid the pandemic spreading of disease. LSPR and SERS-based optical biosensors have already shown their potential in the detection of viral pathogens as SARS-Cov-2 [126,127,128]; for this reason, combining this to wearable biosensors by embedding the transducing elements on a flexible substrate could be a winning strategy to pursue, as reported also by Choe et al. [124] (Figure 6b).

### 4.3. Food Quality Monitoring

Due to the overall increase of the world population in the past decades, avoiding food waste is becoming a fundamental necessity; for this reason, the growing food industry is working on the improvement of the long-storage and preservation of food with novel packaging and delivery systems [53]. In this scenario, the biosensing of freshness markers, pathogens, allergens, and toxic agents in food is evolving toward the so-called smart active packaging [52]. Many sensors have been already proposed for food monitoring, but, again, some of the commonly encountered issues hide in the robustness, selectivity, and sensitivity of the proposed devices. Smart colorimetric labels could provide a “quality index” of the food by simply exhibiting a color variation visible to the naked eye [129]. Even though the implementation of some devices in smart and active packaging has already been proposed, for instance in refs. [53,130], one of the main challenges remains the achievement of a multiplexed sensing of the many different factors affecting the quality of certain food. Flexible optical biosensors have appealing multifunctional capabilities enabling both contaminants detection and longer shelf-life of food due to the sensing mechanisms, herein reported, and to the antimicrobial activity of noble-metal NPs [44,131]. For this reason, the use of polymers combined with optically active nanomaterials exhibit promising potential also in food quality monitoring. A smart application for the SERS-based detection of pesticides in fruits and vegetables has been reported in ref. [125] (Figure 6c), but many other flexible platforms are currently ready for these applications.

## 5. Conclusions and Future Perspectives

Label-free flexible LSPR and SERS-biosensors represent promising platforms for point-of-care testing, wearable biosensors, and direct food quality assessment.

The most appealing qualities of these devices come from their low-cost fabrication, their capability of adapting to non-planar surfaces, and their scalable production. Both synthetic and natural polymers have been extensively employed for the fabrication of flexible biosensors for their appealing physicochemical properties. These properties enable the easy integration of noble-metal nanoparticles within or on the polymeric matrices. Also, if the nanoparticles are conveniently functionalized through active bioprobes, they give rise to highly selective transducers. The shape, size, and material of the nanoparticle can be engineered to achieve high sensitivity and low limits of detection.

In this review article, we summarize the recent advances in the development of flexible optical biosensors. First, LSPR-based biosensors are reviewed and classified in 2D and 3D arrays of nanostructures. Then, flexible SERS-based biosensors are considered, which may be based on either synthetic or natural polymers. Particular attention is paid to transparent polymers since their *in-situ* application is generally easier. The performances of flexible biosensors in terms of stability, sensitivity, early detection, and reversibility are studied. Many fabrication strategies are carefully reviewed, highlighting their application domain, resulting sensitivities, and limits of detection. Although the flexible nanoplasmonics field is relatively recent, many advances have been made to overcome the limitations in the expensive fabrication strategies of rigid platforms. A large variety of flexible platforms has been tested as proofs of concept, but there is still a lot to do in terms of real-life applications. Moreover, although rigid plasmonic platforms generally exhibit high-sensitivity and low limits of detection, they are suitable for none of the applications we have highlighted in this review. However, limits of detection, sensitivity, specificity, and optical response optimization are still an active research field and necessitate strong improvements. For these reasons, due to the exponential growth of the research toward novel flexible optical biosensors of the last years, the possibility of commercializing them can be reasonably considered in the near future.

## Data Availability

Not applicable
